# Leveraging Generative Large Language Models for Temporal Relation Extraction From French Clinical Narratives: Prompt-Chaining Approach

**DOI:** 10.2196/95198

**Published:** 2026-09-30

**Authors:** Nesrine Bannour, Guillaume Assié, Anne Sophie Jannot, Olivia Boyer, Nicolas Garcelon, Xavier Tannier, Marc Vincent

**Affiliations:** 1 Clinical Bioinformatics Laboratory, INSERM UMR 1163, Imagine Institute, Université Paris Cité, Paris, France; 2 Université Paris-Cité, Imagine Institute, Data Science Platform, INSERM UMR 1163, 24 Bd du Montparnasse, Paris, 75015, France; 3 Université Paris Cité, CNRS, Inserm, Institut Cochin, Paris, France; 4 Department of Endocrinology and National Reference Center for Rare Adrenal Disorders, AP-HP, Hôpital Cochin, Paris, France; 5 Banque Nationale de Données de Maladies Rares, Assistance Publique – Hôpitaux de Paris, Paris, France; 6 UMRS 1346 – HEKA, Université Paris Cité, Inserm, Inria, Paris, France; 7 Néphrologie Pédiatrique, Centre de Référence MARHEA, Hôpital Universitaire Necker-Enfants Malades, Assistance Publique - Hôpitaux de Paris (APHP), Imagine Institute, INSERM UMR 1163, Université Paris Cité, Paris, France; 8 Sorbonne Université, Université Sorbonne Paris-Nord, Inserm, Limics, Paris, France

**Keywords:** large language models, electronic health records, temporal relation extraction, information extraction, prompting engineering, clinical text, rare diseases, phenotype

## Abstract

**Background:**

Temporal relation extraction (TRE) in clinical narratives is crucial for understanding patient history, disease progression, and treatment pathways. However, it remains challenging due to limited annotated data and to the complexity of clinical text, including domain-specific terminology, inconsistent information, and implicit temporal reasoning. These challenges are particularly acute for rare diseases, where information such as the date of diagnosis or the onset of key phenotypes is rarely available in structured data, despite being essential for estimating diagnostic delay and reconstructing the natural history of the disease.

**Objective:**

We explore the potential of open-weight and on-premises large language models (LLMs) on clinical TRE and normalization using zero- and few-shot prompting, aiming to reduce the time-consuming and costly annotation process. Based on recent promising capabilities of LLMs in understanding and reasoning over text, we evaluate whether these models can efficiently perform TRE and normalization in real-world clinical settings.

**Methods:**

We cast the temporal relation task as a question-answering task, in which the models extract the temporal expressions associated with a given clinical event. We propose a prompt-chaining strategy that sequentially performs normalization on the extracted temporal expressions. Our LLM-based approach is evaluated on constructed and annotated French real-world clinical narratives, covering temporal relations involving 2 types of clinical events: phenotypes and rare disease diagnoses. Four open-weight LLMs were evaluated across multiple prompt configurations and compared with rule-based and neural baselines using exact-match metrics, an LLM-as-a-judge evaluation, and human validation. We further evaluate our approach by applying it to the English 2012 i2b2 corpus, involving other types of clinical events.

**Results:**

The proposed approach achieves stable extraction performance with only a small number of in-context examples across both clinical event types, reaching a maximum *F*_1_-score of 0.72 for rare disease diagnoses and 0.61 for phenotype events. LLM-as-a-judge evaluation supports these findings by capturing minor temporal variations penalized by exact-match metrics, while human validation further confirms the clinical validity of the extracted relations. On the 2012 i2b2 corpus, the approach remains robust despite increased complexity and minimal supervision. Adding normalization using the prompt-chaining strategy maintains stable overall performance, indicating minimal error propagation and efficient temporal normalization.

**Conclusions:**

Prompting LLMs with a minimal set of in-context examples can achieve strong performance for TRE and normalization in low-resource settings with limited domain-specific annotations. Relying on open-weight, on-premises models, the proposed approach therefore offers a practical path toward deployment in real-world clinical workflows and could further be extended to extract key clinical information, such as age at diagnosis or age at onset, particularly in the context of rare diseases, where it could help prefill structured reporting forms, reducing the manual effort needed to retrieve such information directly from clinical narratives.

## Introduction

### Background and Motivation

Electronic health records are widely regarded as having significant potential to improve clinical research. However, most data contained in electronic health records are in free-text format. Unstructured text contains up to 80% of crucial clinical information, including temporal information [1-3]. Several temporal information extraction approaches have been proposed in recent years to facilitate access to this information, which can help better understand the patient’s prior medical history, disease progression, treatment efficacy, etc. Temporal information extraction includes detecting events, identifying temporal expressions, and extracting temporal relations between them.

In the context of rare diseases, extracting specific temporal information such as the date of diagnosis and the dates of key phenotypes is crucial. Such temporal anchors enable the reconstruction of patient care pathways, support prognostic assessments, and allow researchers to characterize the natural history of rare diseases that are often poorly documented. This information is also required for the curation of rare disease registries and clinical research databases, where structured entries are derived from clinical narratives for reporting and research purposes. In practice, clinicians often need to retrospectively review these narratives to identify and structure relevant temporal information. Automating this process will facilitate the construction of longitudinal patient timelines, improve registry completeness, and reduce the burden of manual reporting.

### Prior Work in Clinical Temporal Relation Extraction

There has been a significant interest in temporal relation extraction (TRE) in the clinical domain through the 2012 i2b2 challenge [4] and the 2015‐2017 Clinical TempEval shared tasks [5-7]. Clinical TRE methods evolved from rule-based [8-10] to feature-based machine learning [11-13] and neural-based methods [14-19].

Early systems relied on manually defined rules [9,10,20], which required human expertise and limited generalizability across domains. Following rule-based systems, a range of feature-based machine learning methods has been developed, using, for instance, support vector machine classifiers and conditional random fields, leveraging a range of syntactic, lexical, and semantic features [11,12,20,21]. Tourille et al [12] evaluated random forest and support vector machine models to tackle TRE for clinical corpora in both English and French. Some research studies introduced a hybrid approach that integrates rule-based techniques with a maximum entropy classifier and evaluated its performance on the 2012 i2b2 dataset [8].

Neural approaches offered significant improvement to clinical TRE, outperforming traditional feature-based models. Early deep learning methods used convolutional neural networks (CNNs) [19,22] and long short-term memory networks [14,17,23,24]. Subsequently, the emergence of pretrained transformer-based models [25] has driven several research efforts on clinical TRE [15,26,27], applied to benchmark datasets such as THYME [28] and 2012 i2b2. Han et al [16] introduced OpenNRE, an open and extensible toolkit that implements neural relation extraction models using diverse encoding architectures, such as CNNs, piecewise convolutional neural networks (PCNNs) [29], and recurrent neural networks. Other studies have further explored the use of graph neural networks for document-level TRE, aiming to model long-range dependencies between events and temporal expressions. For instance, GRAPHTREX was introduced as a hybrid approach combining span-level entity representations with a heterogeneous graph to model both local and global dependencies [30].

### Challenges in Clinical TRE

Despite notable progress, TRE in clinical texts remains challenging. To obtain high-performing supervised temporal relation extractors, large amounts of annotated corpora are required. However, the annotation process is known to be time-consuming and highly expensive, particularly in the clinical domain, due to domain expertise need. Consequently, the available datasets have low interannotator agreement [31], and annotated corpora are limited for non-English languages. Only a few research works have been conducted on French corpora [12,23,32,33]. Moreover, clinical narratives often contain implicit temporal reasoning, domain-specific terminology, and long-distance relations, which continue to limit model performance in real-world applications [34].

### Large Language Models for Clinical TRE

With the emergence of generative large language models (LLMs), showing promising results in a variety of natural language processing (NLP) tasks, a few studies have evaluated their potential on clinical TRE. Generative LLMs, such as BART [35], T5 [36], GPT [37], and Llama models [38], have been used for TRE by casting the task as a text-to-text problem. Different input or output representations impact the performance of encoder-decoder models (T5 and BART) on the Clinical TempEval 2016 dataset, showing that prompting one event at a time yields the best performance [39]. Saiz and Altuna [40] fine-tuned Relation Extraction By End-to-end Language generation [41], a BART-based sequence-to-sequence model originally designed for general TRE, on the 2012 i2b2 corpus, achieving competitive results. Some research work explored the use of ChatGPT in zero-shot and few-shot settings for the TRE task [42,43], reporting that its performance remains limited for long-distance dependencies. A recent review study [44] stated that generative LLMs are not widely used for clinical TRE. Furthermore, most efficient models are offered as privately owned services, limiting their use with sensitive data. The advent of open-weight models, most notably the Llama model family [38], has facilitated the use of LLMs on real-world data. A new generation of built-in reasoning LLMs has been released, such as DeepSeek-R1 [45] and Qwen3 [46], to enhance complex task solving.

Research efforts on open-weight LLMs for clinical TRE showed that zero-shot performance is generally lower than supervised approaches and that temporal consistency remains challenging [47]. An LLM-based method combining automated prompt optimization with few-shot in-context learning to predict temporal relations [48] was proposed in the 2024 ChemoTimelines shared task [49], noting that although fine-tuned smaller LLMs perform best, few-shot prompting can produce reasonable results in low-resource scenarios. Recently, Andrew et al [33] evaluated LLMs for extracting temporal relations from French pediatric clinical reports, showing that few-shot prompting with simplified binary labels outperforms multiclass approaches. Their results highlight the potential of LLMs for clinical timeline construction while handling sensitive data securely.

Latest research has examined prompt-tuning and prompt-learning as efficient alternatives to full fine-tuning in low-resource clinical relation extraction [50-52], though only a few have focused on clinical TRE [53]. An instruction fine-tuning method obtained the highest results [54] in the 2024 ChemoTimelines shared task, using the Flan-T5 model [55]. For the 2025 edition [56], Zhao and Vydiswaran [57] combined supervised fine-tuning of Llama models for temporal relation classification with zero-shot Qwen3 prompting for normalization and rule-based postprocessing, obtaining a second-place ranking. As described by Zaghir et al [58], there is a significant lack of open-source and locally deployable LLM-based approaches for real-world clinical applications, particularly for French.

### Contributions

In this paper, we use open-weight on-premises generative LLMs to extract temporal relations from French clinical narratives in zero-shot and few-shot settings, relying solely on in-context learning [59], that is, the ability of the model to learn from directly embedded instructions and examples in the prompt at inference time. Our study focuses on low-resource scenarios, where only few annotated examples are available. Our main goal is to extract the temporal expressions associated with clinical events. To this end, we cast the TRE task as a question-answering problem.

The main contributions of this paper are summarized as follows:

- We propose an LLM-based zero- and few-shot prompting approach that recasts TRE as question answering, identifying the temporal expressions associated with a given clinical event. The extracted expressions are then normalized to a standardized format using a prompt-chaining strategy. To build and evaluate our LLM-based approach, we construct and annotate a real-world corpus of clinical reports written in French, covering temporal relations for phenotypes and rare disease diagnoses, using a defined annotation guideline.
- We evaluate 4 open-weight on-premises LLMs, including built-in reasoning models, of comparable parameter sizes in both zero-shot and few-shot settings, comparing them to a rule-based baseline method and neural methods based on the OpenNRE framework.
- We assess the performance of our approach using exact-match extraction metrics and go beyond them by incorporating LLM-as-a-judge evaluation to capture subtle temporal variations, complemented with human validation to ensure clinical correctness.
- We further evaluate our method on the publicly available English 2012 i2b2 dataset to enable comparison with subsequently developed methods and to assess its robustness for extracting temporal information from clinical narratives in diverse clinical settings.

## Methods

### Task Formulation

TRE involves identifying and classifying the relation between pairs of events, pairs of temporal expressions, or between an event and a temporal expression. In this work, we are interested in extracting the temporal relations between events and temporal expressions. As illustrated in the left panel of Figure 1, traditionally, the task is addressed by first constructing all possible event-temporal expression pairs, selecting relevant candidate pairs, and then classifying the relation between each pair. As shown in the right panel of Figure 1, we reformulated this task as a question-answering problem. Given a clinical text and a clinical event, the objective is to identify and extract all temporal expressions within the text that are associated with the event. If no such temporal expressions exist, the model should return “None”. Our aim with this task definition and the focus on a single relation type is to develop a reproducible and practical method suitable for real-world clinical use. This reformulation combines the selection of relevant temporal expressions and relation identification into a unified task. Moreover, we used a prompt-chaining strategy to perform normalization on the extracted temporal expressions.

**Figure 1.**
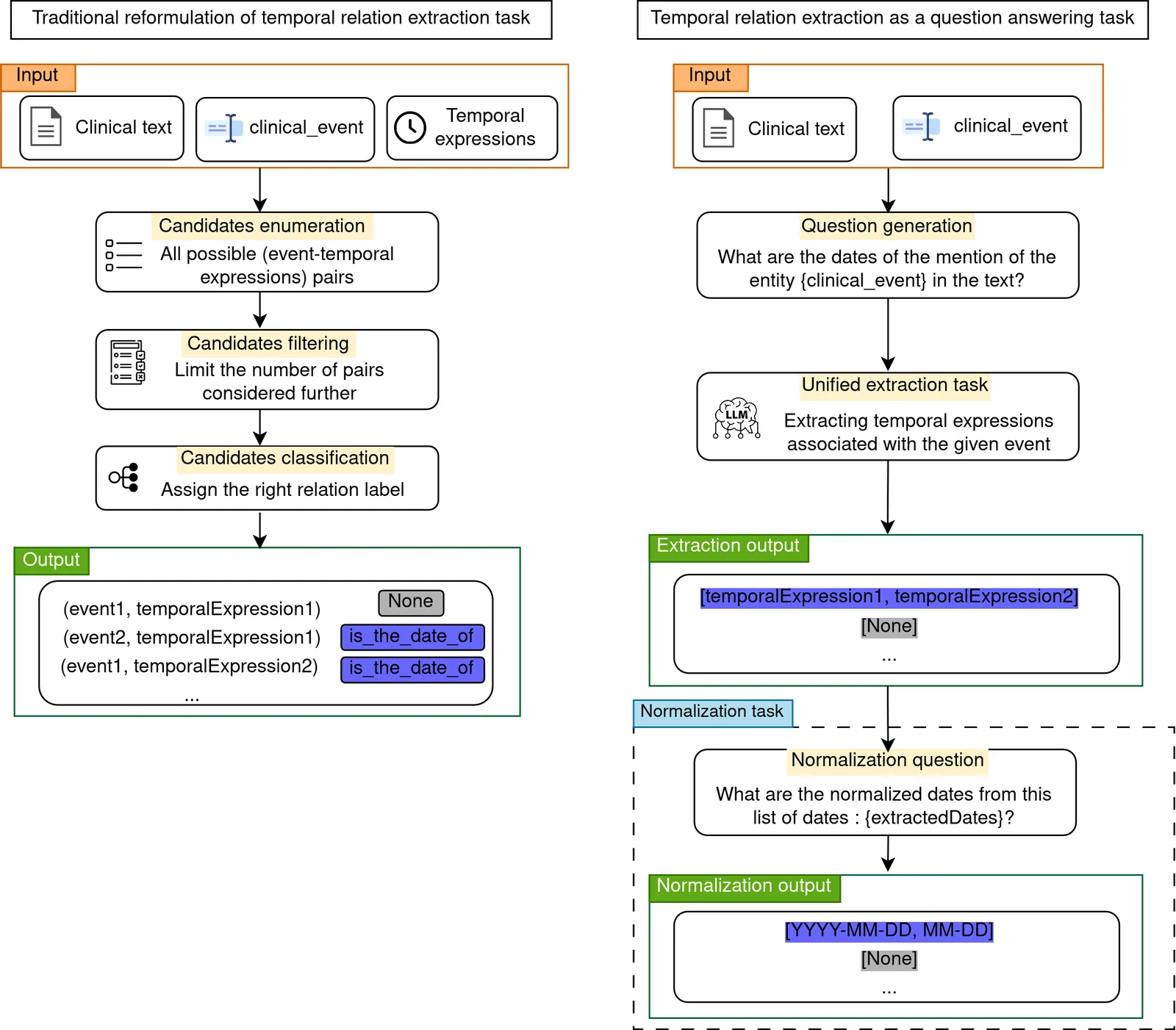
Temporal relation extraction: traditional formulation (left) versus our question-answering reformulation with normalization step (right). The left panel depicts the traditional pipeline of candidates’ enumeration, filtering, and classification. The right panel illustrates our reformulation, where a question derived from a clinical event is used to query a large language model to extract all associated temporal expressions. The dashed box indicates the normalization step.

### Prompt Design

#### Overview

The prompt design follows a sequential prompt-chaining framework with 2 distinct prompts: one for temporal extraction and a second for normalizing the extracted expressions. Temporal normalization refers to the process of converting temporal expressions in text into a standardized format, such as YYYY-MM-DD. The same LLMs were used for both steps, forming a pipeline in which the outputs of the extraction prompt are fed directly into the normalization prompt.

#### Step 1: Temporal Extraction

The LLMs were prompted in French to answer a standardized question for each clinical event: “What are the dates of the mention of the entity {clinical_event} in the text?”

The expected output was either a list of temporal expressions or a list containing “None” when no date is associated with the queried event. As shown in Figure 2 (English version of the prompt), each prompt is organized into 3 components: a task description, specifications for a structured output, and a set of explicit instructions. We evaluated the following 4 prompt configurations:

- Text: the model is prompted with the full clinical text and the associated question, without any guidance.
- Text+rationale: the model is prompted with the same input as in the Text configuration but is additionally instructed to generate 1 or 2 sentences of rationale before producing the final answer.
- Text+givenDates: in addition to the input used in the Text configuration, the model is provided with the list of all temporal expressions mentioned in the document (including “None”), obtained through a rule-based extraction method with additional human review, and is instructed to limit its answer on this list.
- Text+rationale+givenDates: the model receives the same inputs as in the Text+givenDates configuration and is further instructed to generate a brief rationale prior to the generation of the final answer.

**Figure 2.**
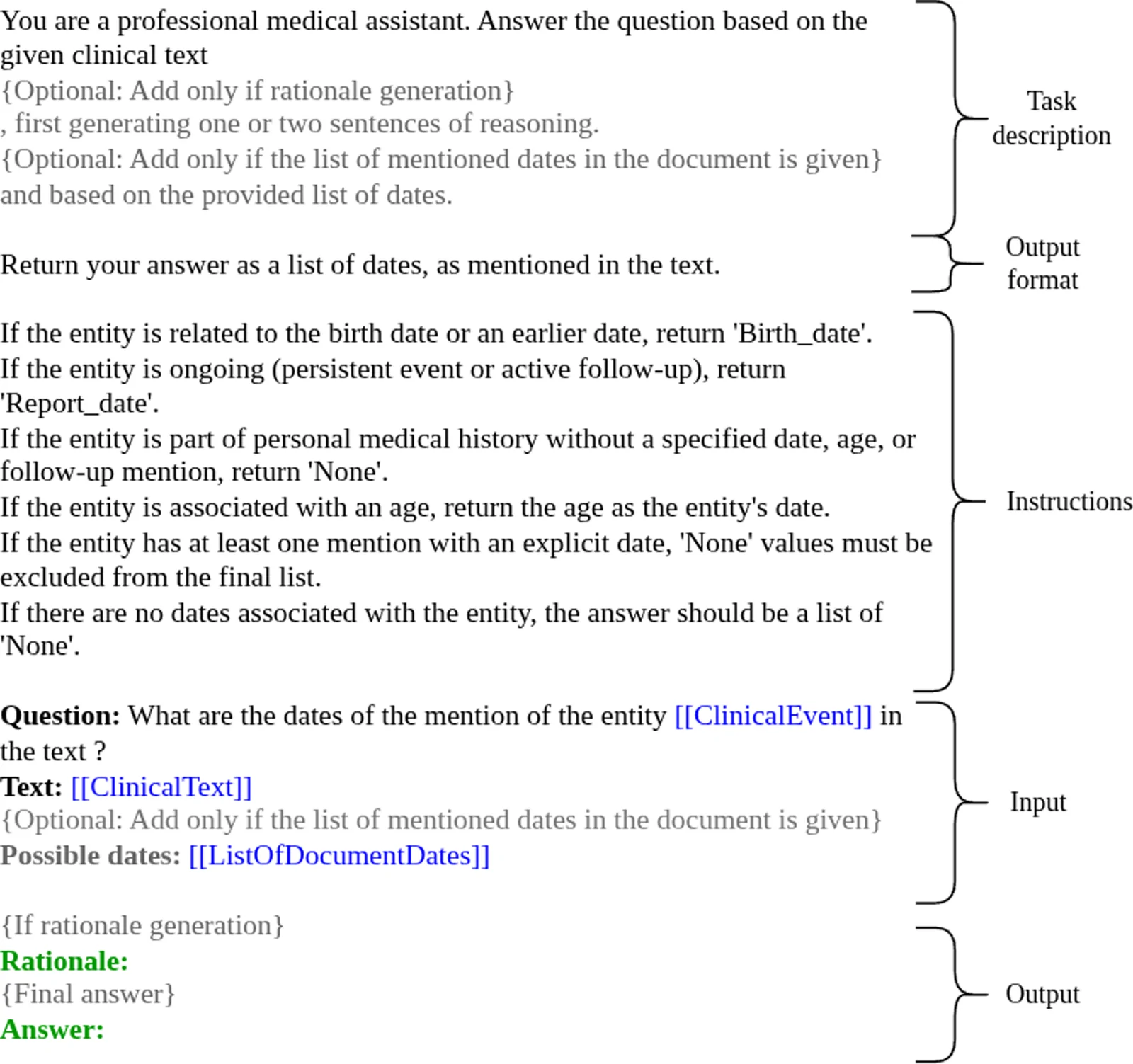
The main structure of the extraction prompt.

#### Step 2: Temporal Normalization

The LLMs were prompted to normalize the list of extracted temporal expressions from the step before. Temporal normalization is challenging due to ambiguous temporal expressions, relative dates such as “three weeks ago” or “in two days” that require a reference time, and diverse formats. In our work, the LLMs were prompted to normalize relative dates using the report date. Similar to step 1, the normalization prompt is composed of different components to ensure consistent normalization of the extracted temporal expressions into the format YYYY-MM-DD, as illustrated in Figure 3.

**Figure 3.**
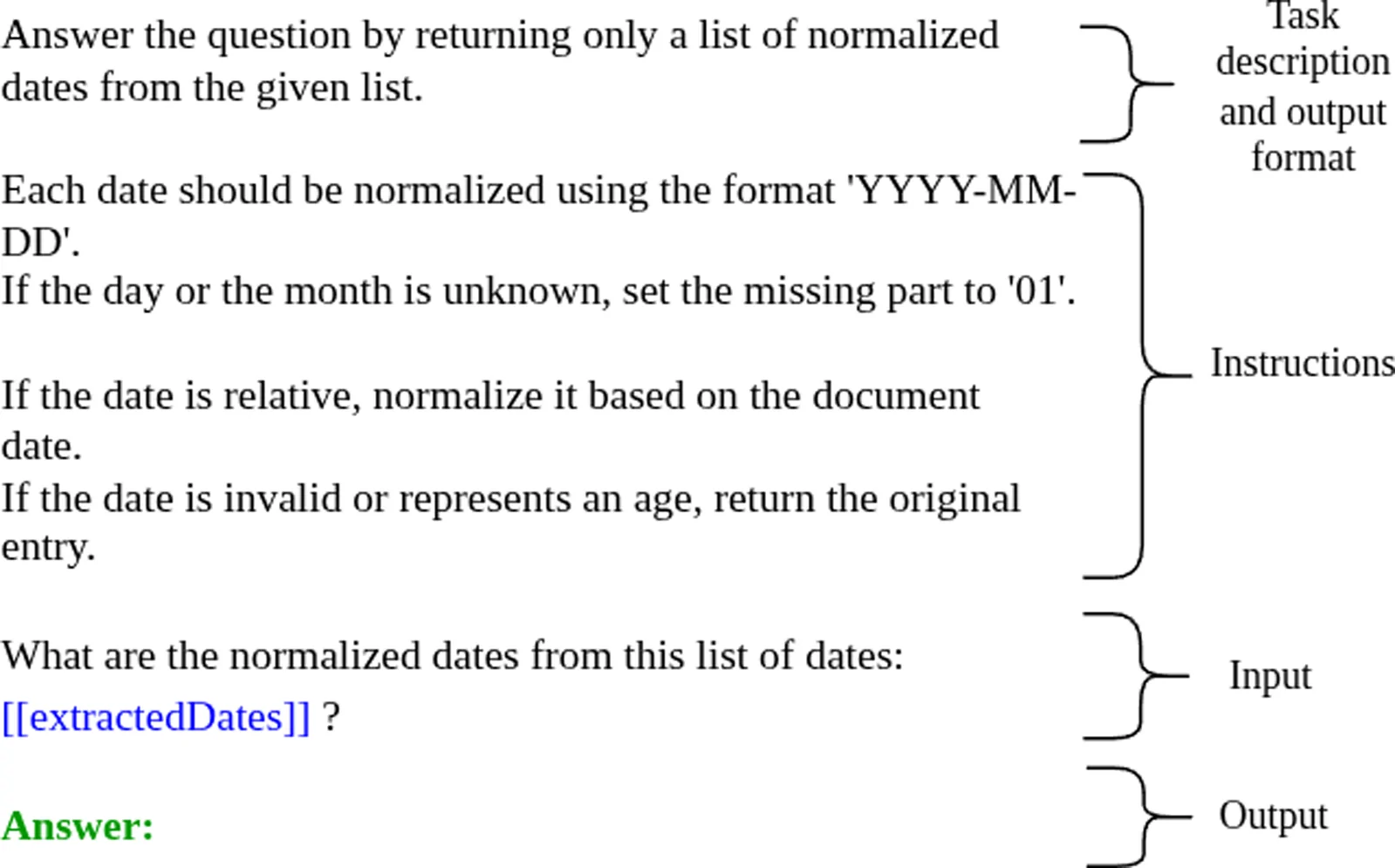
The main structure of the normalization prompt.

We applied the same model sequentially to the extraction and normalization steps, resulting in a prompt-chaining pipeline that preserves dependencies between tasks. In the few-shot setting, a fixed number of annotated examples were included as in-context demonstrations for each subtask. The number of demonstrations was determined through iterative prompt refinement on the development set, progressively adding examples based on error analysis and targeting challenging cases such as long-range event-temporal dependencies and ambiguous temporal references, until performance reached a satisfactory level. This resulted in 14 demonstrations for phenotype events, 6 demonstrations for rare disease diagnoses in the extraction prompt, and 6 demonstrations for both event types in the temporal normalization prompt. These demonstrations were shared across all models and input configurations within each task.

### Dataset

#### Corpus Construction

Our work was done in the context of the C’IL-LICO research project on ciliopathies. This project and its study protocol were approved by the French National Ethics and Scientific Committee for Research, Studies and Evaluations in the Field of Health (approval 2201437). The data processing was approved by the French Data Protection Authority (CNIL) with a waiver of informed consent under number DR-2023‐017//920398v1.

We constructed a corpus of 85 deidentified French patient clinical reports (hospitalization and consultation notes) extracted from Dr. Warehouse, the French clinical data warehouse at Necker Hospital [60]. Clinical reports were chosen to ensure sufficient representation of the 2 clinical events of interest: rare disease diagnoses and phenotypes. We annotated 22 documents to create the prompt examples, 21 documents for validating and refining the prompting strategy, and 42 test documents for final evaluation of our LLM-based approach.

#### Clinical Events and Temporal Expression Annotation

Clinical events were initially preannotated using a dictionary-based approach with exact string matching between the clinical text and a terminology-driven list of terms. Rare disease diagnoses were annotated using a filtered Orphanet-based dictionary derived from the 2023 French Orphanet nomenclature file [61], excluding diseases classified as nonrare in Europe, obsolete disease names, and terms corresponding to pathology groups. Phenotypes were annotated using a custom dictionary built from the French translation of the Human Phenotype Ontology [62] and its synonyms, mapped to the Unified Medical Language System concepts and filtered to retain only Disorders semantic group terms from the 2023AA release [63], which includes semantic types relevant to diseases, abnormalities, and clinical findings. Both the Orphanet- and Human Phenotype Ontology–based dictionaries were further filtered to exclude terms shorter than 4 characters to reduce noise. The used dictionary-based approach was built using the Entrepôt de données de santé-natural language processing (EDS-NLP) framework [64], which includes built-in support for contextual qualifiers such as negation, hypothesis, and family history. The resulting preannotations were subsequently manually validated by 2 medical experts, who corrected erroneous matches and added missing terms. Overall, as shown in Table 1, a total of 488 phenotype mentions and 81 rare disease mentions were annotated. Temporal expressions were detected and normalized using the eds.dates [65] component of EDS-NLP. For relative temporal expressions, normalization was performed using the document date, which is encoded in the clinical report filename as recorded in the data warehouse.

**Table 1.** Test data statistics per type of event.

Event type	Documents, n	Event mentions, n	Relations, n	“Doctime” relations, n	“None” relations, n
Phenotype	42	488	356	163	83
Rare disease	42	81	70	40	4
Clinical concept (i2b2)	120	9767	10,087	1860	4079

#### Temporal Relation Annotation

In addition to clinical events and temporal expressions, we manually annotated the document date for each report, defined as the consultation date, admission date, or the report’s writing date when the former are unavailable. In specific cases, such as day hospital admission reports introduced by expressions like “Day hospital admission report” followed by “Treatment session on,” the document date is defined as the date indicated by the treatment session, as the described events are reported relative to it. Temporal relations are annotated by assigning an “is_the_date_of” relation between a clinical event and an associated temporal expression when such a relation is present. Preannotated temporal expressions and their normalized values are manually corrected only when involved in an annotated temporal relation, while all other temporal expressions remain unchanged. Table 1 presents the number of annotated relations for rare disease diagnoses and phenotype events. It includes relations linking events to the document date (“Doctime” relations) and “None” relations, which represent cases where no temporal expression is associated with any mention of the event.

For events with multiple mentions, only the mentions linked to temporal expressions are retained, which is reflected in the counts reported in Table 1. Annotation was conducted using the BRAT annotation tool [66]. Negated event mentions and family-related events are not included and are therefore not considered when querying the LLMs.

#### Adaptation of the 2012 i2b2 Corpus

For comparison, we evaluated our TRE approach on the 2012 i2b2 corpus [4,67], which includes 310 discharge summaries written in English, split into a training and validation set of 190 documents and a test set of 120 documents. This corpus was annotated with 4 types of events, 4 types of temporal expressions, and 7 types of temporal relations, which are merged into 3 main categories: BEFORE, OVERLAP, and AFTER. We adapted the corpus to our real-world setting through several preprocessing steps. We first inferred all transitive temporal relations using the challenge’s official evaluation scripts and restricted the annotations to event-temporal expression relations. We then retained only clinical concepts (problems, tests, and treatments), excluded duration and frequency temporal expressions, and selected the temporal relation types most consistent with our annotation strategy. Specifically, we kept the BEFORE_OVERLAP, ENDED_BY, and BEGIN_BY relations, and the OVERLAP category, which includes SIMULTANEOUS, OVERLAP, and DURING relations. All relations were renamed “is_the_date_of” as our initial corpus. Table 1 describes the number of retained relations in the test set.

To apply our approach to the i2b2 corpus, all prompts were translated into English, and 10 in-context demonstrations sampled from the training set were used in the few-shot setting. The evaluation focused exclusively on the TRE and does not include the normalization of the extracted temporal expressions. Supplementary details on the character-based distances between clinical events and their associated temporal expressions in the test set are provided in Table S1 in Multimedia Appendix 1.

### Language Model Configurations

We evaluated 4 generative LLMs, namely, Llama3.3 with 70.6 billion parameters, Qwen2.5 with 72.7 billion parameters, Qwen3 with 32.8 billion parameters, and DeepSeek-R1 with 32.8 billion parameters, all hosted on an on-premises Ollama server to ensure data privacy. Models were served in an 8-bit quantized format for efficiency without compromising performance, and a default context length of 32,000 tokens is used for all experiments. To minimize the variance in outputs, we used a temperature parameter of 0. For the built-in reasoning models Qwen3 and DeepSeek-R1, the maximum number of generated tokens was limited to 2000 to prevent overly long reasoning.

### Baseline Methods

We compare the performance of our LLM-based approach with 2 baseline methods: a proximity-based relation extraction method [68] and the deep learning–based OpenNRE relation extraction toolkit [16]. The following section presents some details regarding these 2 baseline approaches.

#### Proximity-Based Relation Extraction Method

This method is a heuristic approach that infers relations based on the textual distance between entities, assuming that closer entities in the text are more likely to be related. It was originally developed to link medications with their associated attributes, where a source entity (eg, a medication) may be associated with multiple target entities (its attributes), while each target entity is generally linked to a single source entity. This assumption is less strictly applicable in the context of TRE.

In our work, we adopted the symmetric proximity method, which considers distances in both directions and links each target to the nearest source, making it better suited to the variable placement of temporal expressions in clinical narratives. The maximum distance was empirically set to 600 characters, without sentence-level restrictions due to ambiguous sentence boundaries in clinical reports.

#### OpenNRE-Based Method

This method is an open-source and extensible toolkit for neural relation extraction. It classifies the type of relation between a pair of target entities in a sentence. OpenNRE supports a wide range of neural architectures, including CNN-, recurrent neural network-, and transformer-based models. Although originally designed for sentence-level extraction, we adapt it to clinical narratives by relaxing the sentence constraint and considering broader contexts around event-temporal expression pairs. The goal is to predict whether an “is_the_date_of” or “None” relation exists between each event and each temporal expression. We trained several models using different encoders, including CamemBERT, ModernCamemBERT, and CNN-based encoders (CNN and PCNN). For transformer-based encoders, we explored the two available encoder representation strategies: (1) using the special classification [CLS] token embedding to represent the input (CLS-based) and (2) using the embeddings of the entity start positions to represent the entities (entity-based).

For each encoder and representation strategy, we trained 4 models corresponding to different input configurations:

- allPairs+FullText: The full clinical text is provided as input for each pair, and all possible candidate event-temporal expression pairs are considered.
- allPairs+Passage: Only the passage between the candidate event and temporal expression is provided as input, while still considering all candidate pairs.
- LimitedPairs+FullText: The full text is provided for each pair, but candidate pairs are restricted to those within 2 maximum distances determined from the training set: 930 characters for relations between events and the document date, and 8070 characters for relations between events and other temporal expressions. This strategy reduces the number of “None” relations in the training set from 2840 to 1188 (approximate reduction of 58.2%) while preserving all “is_the_date_of” relations, thereby alleviating class imbalance. More details on the character-based distances between clinical events and their associated temporal expressions in the train set are provided in Table S2 in Multimedia Appendix 1.
- LimitedPairs+Passage: Only the passage between the event and temporal expression is used, and candidate pairs are similarly restricted by the same 2 maximum distances.

All models were trained jointly on relations involving both rare disease diagnoses and phenotypes and evaluated on the complete set of candidate pairs in the test set to assess their performance in extracting relations between clinical events and temporal expressions.

### Evaluation Metrics

We evaluated our methods using the 3 main information extraction metrics: precision, recall, and *F*_1_-score, focusing exclusively on the positive class (cases where a date is present, as opposed to “None”). Predicted dates were compared to gold-standard dates using exact match after lowercasing. Certain answers were allowed according to the prompt instructions: Report_date was counted as correct if it matches the document date, and Birth_date was considered correct for age- or birth-related events. 95% CIs were estimated using the empirical bootstrap method [69]. Each test corpus is sampled with replacement 1000 times, and the evaluation metrics were computed for each sample.

Evaluating LLMs remains challenging due to outputs with multiple valid interpretations, factual inconsistencies, and variability in structured tasks such as information extraction, which standard metrics often fail to capture [70,71]. Therefore, in addition to this strict evaluation, we performed an LLM-as-a-judge validation to account for minor variations in extracted temporal expressions. We prompted the gpt-oss:20b [72] model as an impartial temporal evaluator. For each clinical event, the model receives the clinical text, the list of predicted dates, and the reference dates. Each predicted date is judged as correct if it matches or is equivalent to a reference date at the same level of temporal precision (year, month, or day), and incorrect otherwise. Precision, recall, and *F*_1_ are then derived from these judgments. This evaluation framework enables scalable and context-aware validation while leveraging the original clinical narrative.

Finally, we conducted a human-based validation on our best-performing experiments to provide a more relaxed assessment of the LLM outputs. We reviewed each predicted temporal relation and assigned a binary judgment (correct or incorrect) based on the clinical text. This precision metric measures the proportion of model predictions that are true positives among all predicted relations.

Note that the normalization step was only evaluated under strict exact-match metrics.

Carbon footprint in terms of CO_2_ equivalent of our LLM-based experiments was estimated using GreenAlgorithms (version 3.0; University of Cambridge) [73], based on runtime, computing hardware, and location, covering the full extraction and normalization pipeline for our real-world corpus and the extraction stage only for the i2b2 corpus.

### Ethical Considerations

This work is part of the C’IL-LICO research project on ciliopathies. This project and its study protocol were approved by the French National Ethics and Scientific Committee for Research, Studies and Evaluations in the Field of Health (approval 2201437). The data processing was approved by the French Data Protection Authority (CNIL) with a waiver of informed consent under number DR-2023‐017//920398v1. The clinical data used in our study were deidentified in accordance with General Data Protection Regulation protocols and regulations, and all our experiments were conducted locally on secure machines. The 2012 i2b2 corpus is available for research purposes under a data use agreement.

## Results

### LLM-Based TRE Performance

Tables 2 and 3 report the exact-match performance with 95% bootstrap CIs across all models, prompting strategies, and input configurations for rare disease diagnoses and phenotype events, respectively. Few-shot prompting consistently outperforms zero-shot extraction for rare disease diagnoses, while for phenotype events, the gains are generally modest or inconsistent across models and input settings.

**Table 2.** Exact-match evaluation of zero-shot and few-shot relation extraction performance (rare disease): precision, recall, and *F*_1_-score.

	Llama3.3			Qwen2.5			Qwen3			DeepSeek-R1		
	Precision (95% CI)	Recall (95% CI)	*F*_1_-score (95% CI)	Precision (95% CI)	Recall (95% CI)	*F*_1_-score (95% CI)	Precision (95% CI)	Recall (95% CI)	*F*_1_-score (95% CI)	Precision (95% CI)	Recall (95% CI)	*F*_1_-score (95% CI)
Zero-shot extraction												
Text	0.34 (0.23‐0.51)	0.67 (0.57‐0.77)	0.45 (0.33‐0.59)	0.61 (0.49‐0.73)	0.67 (0.55‐0.78)	0.64 (0.53‐0.74)	0.16 (0.12‐0.22)	0.61 (0.46‐0.74)	0.25 (0.19‐0.33)	0.19 (0.08‐0.44)	0.55 (0.42‐0.67)	0.29 (0.14‐0.52)
Text+rationale	0.35 (0.26‐0.47)	0.77 (0.67‐0.87)	0.48 (0.38‐0.59)	0.55 (0.43‐0.7)	0.57 (0.45‐0.69)	0.56 (0.46‐0.67)	0.48 (0.38‐0.61)	0.51 (0.4‐0.63)	0.5 (0.4‐0.6)	0.55 (0.42‐0.66)	0.56 (0.41‐0.67)	0.56 (0.42‐0.65)
Text+givenDates	0.38 (0.26‐0.56)	0.7 (0.59‐0.8)	0.49 (0.37‐0.63)	0.61 (0.5‐0.74)	0.7 (0.59‐0.79)	0.65 (0.55‐0.75)	0.3 (0.23‐0.4)	0.77 (0.67‐0.87)	0.43 (0.34‐0.53)	0.13 (0.09‐0.17)	0.92 (0.86‐0.98)	0.22 (0.17‐0.29)
Text+rationale+givenDates	0.42 (0.32‐0.57)	0.74 (0.64‐0.84)	0.54 (0.43‐0.65)	0.69 (0.57‐0.81)	0.67 (0.56‐0.77)	0.68 (0.57‐0.78)	0.65 (0.54‐0.79)	0.57 (0.47‐0.69)	0.61 (0.51‐0.71)	0.72 (0.59‐0.84)	0.57 (0.47‐0.7)	0.64 (0.53‐0.74)
Few-shot extraction												
Text	0.41 (0.31‐0.52)	0.85 (0.74‐0.92)	0.55 (0.44‐0.65)	0.58 (0.47‐0.72)	0.82 (0.73‐0.9)	0.68 (0.59‐0.78)	0.48 (0.36‐0.61)	0.7 (0.59‐0.81)	0.57 (0.46‐0.67)	0.46 (0.34‐0.61)	0.7 (0.6‐0.8)	0.56 (0.44‐0.68)
Text+rationale	0.56 (0.41‐0.72)	0.73 (0.61‐0.81)	0.63 (0.51‐0.73)	0.72 (0.63‐0.82)	0.73 (0.63‐0.83)	*0.72 (0.65‐0.79)* ^a^	0.74 (0.62‐0.87)	0.61 (0.49‐0.72)	0.67 (0.56‐0.77)	0.76 (0.6‐0.82)	0.64 (0.47‐0.72)	*0.7 (0.54‐0.75)*
Text+givenDates	0.32 (0.21‐0.49)	0.68 (0.59‐0.79)	0.44 (0.32‐0.58)	0.53 (0.42‐0.67)	0.71 (0.61‐0.82)	0.61 (0.5‐0.72)	0.6 (0.49‐0.71)	0.79 (0.69‐0.89)	*0.68 (0.58‐0.77)*	0.32 (0.24‐0.43)	0.68 (0.58‐0.78)	0.43 (0.35‐0.54)
Text+rationale+givenDates	0.76 (0.65‐0.87)	0.71 (0.61‐0.81)	*0.73 (0.64‐0.83)*	0.57 (0.44‐0.73)	0.79 (0.69‐0.88)	0.66 (0.56‐0.78)	0.6 (0.42‐0.85)	0.62 (0.52‐0.73)	0.61 (0.49‐0.75)	0.75 (0.62‐0.88)	0.54 (0.43‐0.66)	0.63 (0.52‐0.74)

a For each model, the highest *F*_1_-score across input configurations is highlighted in italics format.

**Table 3.** Exact-match evaluation of zero-shot and few-shot relation extraction performance (phenotypes): precision, recall, and *F*_1_-score.

	Llama3.3			Qwen2.5			Qwen3			DeepSeek-R1		
	Precision (95% CI)	Recall (95% CI)	*F*_1_-score (95% CI)	Precision (95% CI)	Recall (95% CI)	*F*_1_-score (95% CI)	Precision (95% CI)	Recall (95% CI)	*F*_1_-score (95% CI)	Precision (95% CI)	Recall (95% CI)	*F*_1_-score (95% CI)
Zero-shot extraction												
Text	0.44 (0.38‐0.5)	0.77 (0.71‐0.81)	0.56 (0.5‐0.61)	0.48 (0.43‐0.53)	0.75 (0.69‐0.8)	0.58 (0.53‐0.64)	0.2 (0.13‐0.29)	0.66 (0.6‐0.72)	0.31 (0.22‐0.4)	0.24 (0.2‐0.28)	0.5 (0.44‐0.56)	0.32 (0.28‐0.37)
Text+rationale	0.44 (0.38‐0.5)	0.75 (0.69‐0.8)	0.55 (0.5‐0.61)	0.5 (0.44‐0.56)	0.65 (0.59‐0.71)	0.56 (0.51‐0.62)	0.44 (0.37‐0.5)	0.49 (0.42‐0.55)	0.46 (0.4‐0.52)	0.5 (0.43‐0.55)	0.57 (0.5‐0.62)	0.53 (0.47‐0.58)
Text+givenDates	0.45 (0.4‐0.5)	0.74 (0.69‐0.8)	0.56 (0.51‐0.61)	0.5 (0.45‐0.55)	0.75 (0.7‐0.8)	0.6 (0.55‐0.65)	0.34 (0.29‐0.39)	0.77 (0.72‐0.82)	0.47 (0.42‐0.52)	0.1 (0.09‐0.12)	0.85 (0.8‐0.89)	0.18 (0.16‐0.2)
Text+rationale+givenDates	0.46 (0.42‐0.51)	0.75 (0.7‐0.8)	0.57 (0.53‐0.62)	0.53 (0.47‐0.59)	0.72 (0.67‐0.77)	*0.61 (0.56‐0.66)* ^a^	0.53 (0.47‐0.59)	0.67 (0.61‐0.71)	*0.59 (0.53‐0.64)*	0.55 (0.48‐0.61)	0.63 (0.58‐0.68)	*0.59 (0.53‐0.64)*
Few-shot extraction												
Text	0.48 (0.42‐0.52)	0.72 (0.65‐0.76)	0.57 (0.51‐0.61)	0.46 (0.41‐0.52)	0.64 (0.58‐0.7)	0.54 (0.48‐0.59)	0.45 (0.39‐0.5)	0.57 (0.51‐0.62)	0.5 (0.44‐0.55)	0.42 (0.35‐0.5)	0.23 (0.18‐0.28)	0.3 (0.24‐0.35)
Text+rationale	0.58 (0.51‐0.64)	0.6 (0.52‐0.65)	*0.59 (0.53-0.64)*	0.54 (0.47‐0.59)	0.65 (0.58‐0.7)	0.59 (0.52‐0.64)	0.59 (0.52‐0.65)	0.55 (0.49‐0.61)	0.57 (0.51‐0.63)	0.57 (0.47‐0.63)	0.41 (0.34‐0.45)	0.48 (0.4‐0.52)
Text+givenDates	0.44 (0.38‐0.49)	0.79 (0.74‐0.83)	0.57 (0.51‐0.61)	0.4 (0.35‐0.45)	0.6 (0.55‐0.67)	0.48 (0.43‐0.54)	0.43 (0.37‐0.5)	0.7 (0.64‐0.75)	0.53 (0.48‐0.59)	0.24 (0.19‐0.29)	0.59 (0.52‐0.64)	0.34 (0.28‐0.39)
Text+rationale+givenDates	0.61 (0.54‐0.67)	0.54 (0.48‐0.6)	0.57 (0.51‐0.62)	0.52 (0.47‐0.59)	0.69 (0.64‐0.75)	0.6 (0.55‐0.65)	0.6 (0.53‐0.66)	0.58 (0.52‐0.63)	*0.59 (0.53‐0.63)*	0.65 (0.57‐0.72)	0.43 (0.36‐0.49)	0.52 (0.45‐0.57)

a For each model, the highest *F*_1_-score across input configurations is highlighted in italics format.

Configurations including rationale generation provide more balanced precision and recall, while those incorporating candidate temporal expressions achieve higher recall values, particularly when combined with rationale generation. In the zero-shot setting, the Text+rationale+givenDates configuration achieves the best results across all evaluated models for both phenotypes and rare disease diagnoses.

For rare disease diagnoses, the best results are obtained by Llama3.3 in the few-shot setting Text+rationale+givenDates configuration, achieving an *F*_1_ of 0.73. Comparable performance is achieved by Qwen2.5, reaching an *F*_1_ of 0.72 in the few-shot Text+rationale configuration. For phenotype events, overall scores are lower. The best *F*_1_-scores are achieved by Qwen2.5 in the zero-shot Text+rationale+givenDates configuration, with an *F*_1_ of 0.61, and in the few-shot setting with the same configuration, reaching an *F*_1_ of 0.6. Llama3.3 and Qwen3 also yield competitive performance, with maximum *F*_1_-scores of 0.59.

Across both event types, the reasoning Qwen3 and DeepSeek-R1 models obtain lower *F*_1_-scores than Qwen2.5 and Llama3.3, with consistently higher recall and lower precision across most configurations.

Figures 4 and 5 illustrate how character-based distance between clinical events and temporal expressions influences extraction performance for rare disease and phenotype events with Qwen2.5 across all input settings. In both figures, the upper panels present *F*_1_-scores computed within each distance bin, where distance bins are defined by percentiles of the distance distribution (Table S1 in Multimedia Appendix 1). The lower panels show the distance distribution using a Gaussian kernel density estimate.

For rare disease relations, *F*_1_-scores are highest at short distances (up to 57 characters: 0.58‐0.73) and progressively decline across medium distance bins (57‐159 and 159‐315 characters: 0.44‐0.73 *F*_1_) with a substantial performance drop at long distances (more than 315 characters: 0.20‐0.57 *F*_1_ and *F*_1_<0.30 for several zero-shot configurations). The Gaussian kernel density estimate curve indicates that most rare disease relations occur within shorter distances, whereas long-distance relations are less frequent but still more challenging.

For phenotype relations, *F*_1_-scores remain relatively stable (0.52‐0.82) across the first 3 distance bins (13‐1097 characters) for most configurations. The configuration Text+rationale+givenDates achieved the highest performance (0.82 of *F*_1_) in both zero-shot and few-shot settings in the shortest distance range (13‐97 characters). Performance progressively declined as distance increased, with *F*_1_-scores dropping to 0.29‐0.36 beyond 1999 characters.

Results obtained using our LLM-as-a-judge validation framework for the TRE are presented in Tables S3 and S4 in Multimedia Appendix 1. This assessment allows for minor variations in predicted temporal expressions while maintaining strict temporal consistency. The results closely align with the exact-match evaluation but consistently yield higher *F*_1_-scores across all models and event types.

To assess the reliability of the LLM-as-a-judge framework, we compare its judgments with human evaluations on a subset of examples. Across all configurations using Qwen2.5, the agreement between the LLM and the human annotator reaches an average *F*_1_ of 0.96 for phenotypes and 1.0 for rare disease events, indicating a very high level of consistency.

Table 4 reports the proportion of predicted dates judged as correct by a human evaluator for rare disease and phenotype events for our best-performing Qwen2.5 experiments. For rare disease diagnoses, precision ranges from 0.65 to 0.85 across all configurations, and for phenotypes, from 0.6 to 0.71. These values exceed the exact-match precision (Tables 2 and 3), confirming a high precision of the extracted relations.

**Figure 4.**
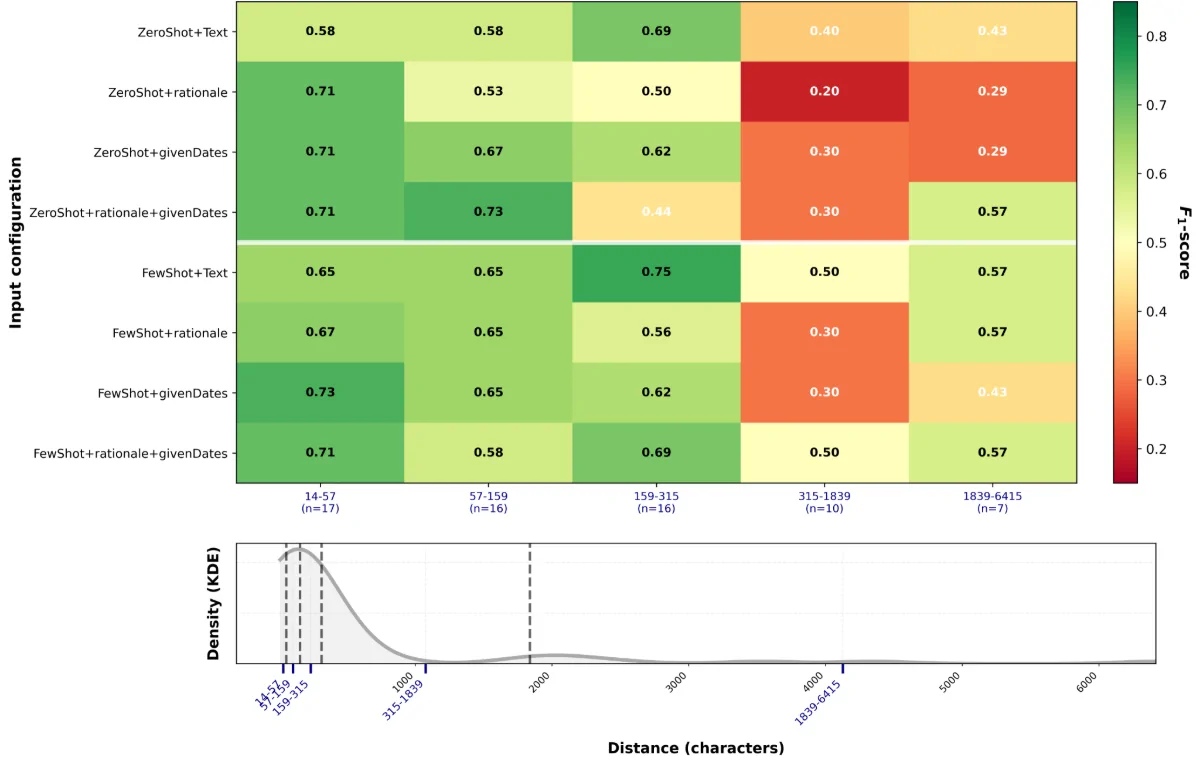
Impact of character-based distance between events and temporal expressions on rare disease relation extraction performance using Qwen2.5. Upper panel: *F*_1_-scores across character-distance bins. The number (n) indicates the number of relations. Lower panel: Gaussian KDE of the distance distribution. KDE: kernel density estimate.

**Figure 5.**
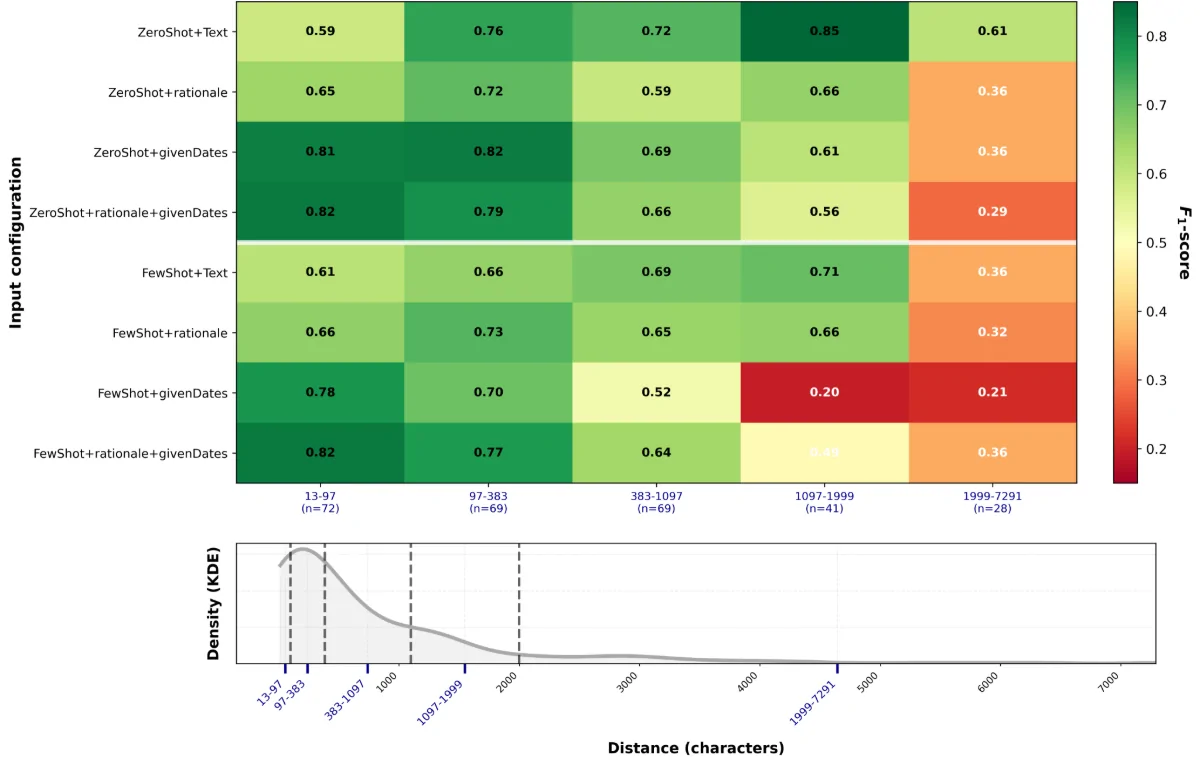
Impact of character-based distance between events and temporal expressions on phenotype relation extraction performance using Qwen2.5. Upper panel: *F*_1_-scores across character-distance bins. The number (n) indicates the number of relations. Lower panel: Gaussian KDE of the distance distribution. KDE: kernel density estimate.

**Table 4.** Human-evaluated precision of model predictions using Qwen2.5^a^.

	Rare disease	Phenotypes
Zero-shot extraction		
Text	*0.85*^b^ (0.61)	0.64 (0.48)
Text+rationale	0.65 (0.55)	0.66 (0.5)
Text+givenDates	0.77 (0.61)	0.61 (0.5)
Text+rationale+givenDates	0.78 (0.69)	0.66 (0.53)
Few-shot extraction		
Text	0.74 (0.58)	0.63 (0.46)
Text+rationale	0.83 (0.72)	0.66 (0.54)
Text+givenDates	0.72 (0.53)	0.6 (0.4)
Text+rationale+givenDates	0.7 (0.57)	*0.71* (0.52)

a Values in parentheses indicate the exact-match precision scores presented in Tables 2 and 3.

b For each model, the highest *F*_1_-score across input configurations is highlighted in italics format.

### Baseline Methods Performance

Table 5 presents the results of the proximity-based relation extraction method on rare disease and phenotype events. The method achieves higher performance on rare disease events, with an *F*_1_ of 0.48, while performance is lower for phenotypes, with an *F*_1_ of 0.24. The values in parentheses in Table 5 correspond to our best-performing LLM, Qwen2.5, which outperforms the proximity-based method across both event types (Tables 2 and 3).

**Table 5.** Exact-match evaluation of proximity-based relation extraction^a^.

	Precision	Recall	*F*_1_-score
Rare disease	0.51 (0.53‐0.72)	0.46 (0.57‐0.82)	0.48 (0.56‐0.72)
Phenotypes	0.31 (0.4‐0.54)	0.19 (0.6‐0.75)	0.24 (0.48‐0.61)

a The ranges indicate the minimum-maximum performance of our best-performing LLM, Qwen2.5, across input configurations.

Table 6 summarizes the performance of the OpenNRE-based models trained on clinical narratives using different encoders (CamemBERT, ModernCamemBERT, CNN, and PCNN), 4 input configurations, and 2 encoder representation strategies for transformer-based encoders, as previously described. Due to the 512-token limit of CamemBERT, which causes truncation of longer clinical texts, we restrict its experiments to passage-based configurations, rather than the full text. Similarly, for the CNN-based encoders, we report results only for the LimitedPairs+Passage configuration.

**Table 6.** Exact-match evaluation of OpenNRE-based relation extraction: precision, recall, and *F*_1_-score.

	AllPairs+FullText			AllPairs+Passage			LimitedPairs+FullText			LimitedPairs+Passage		
	Precision	Recall	*F*_1_-score	Precision	Recall	*F*_1_-score	Precision	Recall	*F*_1_-score	Precision	Recall	*F*_1_-score
Rare disease												
ModernCamemBERT[cls]	0.07	0.64	0.13	0.14	0.72	0.24	0.07	0.75	0.13	0.3	0.55	0.39
ModernCamemBERT[entity]	0.18	0.63	*0.2* ^a^	0.19	0.4	0.26	0.18	0.19	*0.18*	0.16	0.64	0.26
CamemBERT[cls]	—^b^	—	—	0.8	0.46	*0.58*	—	—	—	0.32	0.26	0.29
CamemBERT[entity]	—	—	—	0.8	0.28	0.41	—	—	—	0.65	0.31	*0.41*
CNN-based[cnnEncoder]	—	—	—	—	—	—	—	—	—	0.75	0.04	0.08
CNN-based[pcnnEncoder]	—	—	—	—	—	—	—	—	—	0.21	0.83	0.33
Phenotypes												
ModernCamemBERT[cls]	0.1	0.59	0.1	0.1	0.58	0.18	0.05	0.52	0.09	0.21	0.41	0.27
ModernCamemBERT[entity]	0.01	0.15	*0.12*	0.13	0.34	0.19	0.11	0.17	*0.13*	0.13	0.55	0.21
CamemBERT[cls]	—	—	—	0.57	0.33	*0.42*	—	—	—	0.38	0.28	0.32
CamemBERT[entity]	—	—	—	0.57	0.21	0.3	—	—	—	0.48	0.29	*0.36*
CNN-based[cnnEncoder]	—	—	—	—	—	—	—	—	—	0.71	0.04	0.07
CNN-based[pcnnEncoder]	—	—	—	—	—	—	—	—	—	0.1	0.71	0.17

a For each event type, the highest *F*_1_-score across input configurations is highlighted in italics format.

b Configuration not evaluated.

Across all encoders, CamemBERT achieves the highest *F*_1_-scores on the passage-based configurations (up to 0.42 for phenotypes and 0.58 for rare disease events), while ModernCamemBERT yields lower overall performance, with maximum *F*_1_-scores of 0.27 for phenotypes and 0.39 for rare disease diagnoses. For transformer-based encoders, the [CLS]-based representation generally outperforms the entity-based strategy, particularly for CamemBERT. Results also show that standard CNN-based models achieve high precision (up to 0.75) but very low recall (0.04), while PCNN-based models improve recall (up to 0.83) at the expense of precision (0.1‐0.21).

According to the exact match evaluation (Tables 2 and 3), our best LLM-based configurations outperform the OpenNRE-based models for both rare disease (0.73 vs 0.58 of *F*_1_) and phenotype events (0.61 vs 0.42 of *F*_1_).

### Application on the 2012 i2b2 Clinical Concepts

Table 7 reports exact-match and LLM-as-judge evaluation results for zero-shot and few-shot for TRE on the 2012 i2b2 clinical concepts using Qwen2.5, the best-performing model in our previous experiments.

**Table 7.** Evaluation of zero-shot and few-shot relation extraction (i2b2 clinical concepts): precision, recall, and *F*_1_-score.

	Qwen2.5					
	Exact match			LLM-as-a-judge^a^		
	Precision	Recall	*F*_1_-score	Precision	Recall	*F*_1_-score
Zero-shot extraction						
Text	0.25	0.39	0.31	0.27	0.43	0.34
Text+rationale	0.26	0.35	0.3	0.29	0.38	0.33
Text+givenDates	0.31	0.46	0.37	0.31	0.47	0.38
Text+rationale+givenDates	0.35	0.47	*0.4* ^b^	0.35	0.48	*0.4*
Few-shot extraction						
Text	0.23	0.53	0.32	0.25	0.56	0.35
Text+rationale	0.28	0.46	0.35	0.29	0.49	0.36
Text+givenDates	0.3	0.56	0.39	0.31	0.57	*0.4*
Text+rationale+givenDates	0.32	0.53	*0.4*	0.32	0.54	*0.4*

a LLM: large language model.

b For each evaluation, the highest *F*_1_-score across input configurations is highlighted in italics format.

In the zero-shot setting, exact-match *F*_1_-scores range from 0.30 to 0.40, with the best performance obtained with the Text+rationale+givenDates configuration. Similar results are observed under the LLM-as-a-judge evaluation, with a maximum *F*_1_ of 0.40. In the few-shot setting, performance further improves, with exact-match and LLM-as-a-judge *F*_1_-scores reaching 0.40 for configurations that include the candidate dates, with or without rationale generation. Few-shot prompting consistently increases recall, reaching up to 0.57.

### End-to-End TRE and Normalization Results

Table 8 presents the exact-match evaluation of the end-to-end Qwen2.5 pipeline for TRE and normalization. The extraction component is evaluated under zero-shot and few-shot settings, whereas normalization is consistently performed in a few-shot configuration using 6 examples.

In the zero-shot settings, the highest *F*_1_-scores are obtained when the model uses only the text as input (0.7 for rare disease and 0.62 for phenotypes). In the few-shot setting, performance increases to 0.72 for rare disease, whereas the phenotype score slightly decreases to 0.61, when rationale generation is included. These results indicate that few-shot examples enhance extraction quality for rare disease events while maintaining steady performance for phenotypes. Overall, performance remains stable across event types under the prompt-chaining configuration. The exact-match results for the remaining evaluated LLMs on the end-to-end TRE and normalization pipeline are presented in Tables S5 and S6 in Multimedia Appendix 1.

**Table 8.** Exact-match evaluation of the end-to-end pipeline for temporal relation extraction and normalization.

	Qwen2.5					
	Rare disease			Phenotypes		
	Precision	Recall	*F*_1_-score	Precision	Recall	*F*_1_-score
Zero-shot extraction						
Text	0.67	0.74	0.7	0.51	0.81	*0.62* ^a^
Text+rationale	0.54	0.58	0.56	0.52	0.7	0.6
Text+givenDates	0.59	0.68	0.63	0.46	0.71	0.56
Text+rationale+givenDates	0.69	0.67	0.68	0.5	0.68	0.58
Few-shot extraction						
Text	0.57	0.82	0.67	0.49	0.7	0.58
Text+rationale	0.72	0.73	*0.72*	0.56	0.68	0.61
Text+givenDates	0.52	0.7	0.6	0.38	0.59	0.46
Text+rationale+givenDates	0.56	0.77	0.65	0.49	0.66	0.56

a For each event type, the highest *F*_1_-score across input configurations is highlighted in italics format.

### Environmental Impact

As shown in Table S7 in Multimedia Appendix 1, temporal extraction and normalization inference experiments are estimated to have produced a total of around 21 kg of CO_2_ equivalent, including around 5 kg CO_2_ equivalent from temporal extraction and normalization experiments on the real-world corpus and more than 15 kg CO_2_ equivalent from TRE on the i2b2 corpus using only Qwen2.5.

Among the evaluated LLMs, Llama3.3 had the highest environmental impact (2.3 kg of CO_2_ equivalent), while Qwen2.5 achieved comparable performance on our clinical corpus with a lower carbon footprint (1.5 kg of CO_2_ equivalent). Qwen3 and DeepSeek-R1 produced less than 1 kg of CO_2_ equivalent.

A complete overview of the carbon emissions is provided in Table S8 in Multimedia Appendix 1.

## Discussion

### Prompting Strategies and Model Comparison in TRE

#### Zero-Shot Settings

As reported in Tables 2 and 3, zero-shot prompting demonstrates clear differences in how models handle temporal reasoning using their pretrained knowledge only without prior contextual guidance.

Qwen2.5 consistently achieves the highest *F*_1_-scores across both rare disease and phenotype events. Qwen2.5 and Llama3.3 display less variability in performance across configurations. The slight advantage of Qwen2.5 likely reflects the prompt refinement and example selection performed with this model during development, which enhanced alignment between the task formulation and its reasoning behavior. In contrast, the built-in reasoning models Qwen3 and DeepSeek-R1 show greater variability and lower precision. Their inherent reasoning capabilities occasionally lead to overextended or speculative date predictions, suggesting that unguided reasoning may compromise boundary precision when no contextual examples are provided. However, recall notably increases when candidate date lists are included (0.77 for Qwen3 and 0.92 for DeepSeek-R1 in rare disease extraction). This improvement suggests that explicit temporal cues help these reasoning models anchor their predictions.

#### Few-Shot Setting

Few-shot prompting, even with a limited number of examples, consistently enhances overall relation extraction performance for rare disease diagnoses, though the magnitude of improvement varies considerably by model and input setting. As shown in Table 2 for rare diseases, the gain induced by few-shot prompting makes Llama3.3 the top-performing model overall, with an *F*_1_ rising from 0.54 to 0.73 in the Text+rationale+givenDates configuration. DeepSeek-R1 and Qwen3 benefit the most for initially low-performing configurations (excluding rationale), while Qwen2.5 maintains near-saturated performance. However, phenotype events (Table 3) display inconsistent and limited gains across models and configurations, revealing fundamental task complexity differences.

#### Impact of Textual Distance on Rare Disease and Phenotype Extraction

The impact of textual distance on extraction performance using Qwen2.5 is shown in Figures 4 and 5, with supplementary test data statistics provided in Table S1 in Multimedia Appendix 1. Rare disease relation extraction demonstrates strong performance when events and temporal expressions are closely aligned (up to 0.73 at 14‐57 distance range). This could be due to disease mentions occurring early in the clinical narratives, within the initial presentation or history section, where they are closely aligned with nearby temporal expressions or the document date. Figure 4 confirms this pattern, illustrating that with Qwen2.5, we observe better performance when rare disease mentions are located closer to their associated temporal expressions across all input configurations, and that extraction performance degrades when handling long-range dependencies (more than 315 characters). Additionally, each document generally contains fewer distinct rare disease entities, which reduces the relation extraction complexity and allows a strong overall performance with only 6 in-context examples. In contrast, as shown in Figure 5, phenotype relation extraction exhibits greater robustness across distance ranges, with *F*_1_ ranging from 0.52 to 0.82 for distances less than 1097 characters and from 0.20 to 0.85 within the 1097‐1999 distance range. This difference aligns with the underlying data distribution: rare disease relations are concentrated at short distances, while phenotype relations are more evenly distributed. Providing 14 in-context examples covering both short-range and long-distance temporal links during prompting allows the model to encounter diverse distance scenarios, likely supporting its relative stability across distance ranges. However, even when few-shot prompts include explicit instructions to identify temporal references, such as “Report_date,” extraction performance drops in the highest percentile bins. This indicates that while prompt design can partially mitigate the challenge, it cannot fully overcome the inherent difficulty of modeling long-range event-temporal dependencies.

#### Impact of Input Configurations

Input configuration strongly affects extraction performance. Raw text alone tends to favor recall at the expense of precision, whereas adding rationale generation improves both precision and *F*_1_. Providing candidate dates primarily increases recall but may reduce precision, as seen with DeepSeek-R1 in the zero-shot setting. Combining rationales with candidate dates produces the most balanced results, achieving the highest *F*_1_-scores for rare diseases (0.70) and phenotypes (up to 0.61), underscoring the benefit of guided reasoning alongside temporal cues. These trends are generally supported by the CIs, with some variability across models and configurations.

#### LLM-as-a-Judge Evaluation

Tables S3 and S4 in Multimedia Appendix 1 report the extraction performance using an LLM-as-a-judge validation framework, allowing for minor temporal variations that strict exact-match metrics would penalize. Overall, this validation approach yields higher *F*_1_-scores across models and input configurations, likely reflecting a more realistic assessment of semantically correct but format-divergent outputs, as confirmed by the strong agreement with the human judgment. Qwen2.5 achieved the highest *F*_1_-scores in the zero-shot setting, reaching 0.74 for rare disease diagnoses and 0.62 for phenotypes. However, some errors persist in this evaluation process. For instance, a prediction of “10/12/2020” for a range “10 au 12/12/2020” may still be marked incorrect, while a relative expression such as “ce jour” (ie*,* “that day”) may be incorrectly validated when it refers to a previous consultation or hospitalization rather than the current document date. Despite these limitations, as noted earlier, a strong agreement with human judgment is observed when evaluating Qwen2.5 experiments, supporting the overall reliability of this evaluation approach.

### Comparison With Rule-Based and Neural Baselines

We compare our LLM-based approach with a proximity-based rule baseline and supervised OpenNRE-based neural models. The proximity baseline achieves an *F*_1_ of 0.48 for rare disease diagnoses and 0.24 for phenotypes (Table 5), indicating that rare disease mentions are often temporally anchored by nearby expressions, whereas phenotype-temporal relations are more dispersed and varied, resulting in low recall. Several LLM configurations, including in zero-shot settings, substantially outperform the proximity baseline for phenotypes, with Qwen2.5 and Llama3.3 reaching *F*_1_-scores of 0.55‐0.61 (gains of 31‐37 points). For rare disease relations, most zero-shot configurations also already outperform the proximity baseline, achieving up to 0.68 of *F*_1_ (Qwen2.5, Text+rationale+givenDates), and only 6 few-shot examples are sufficient to exceed 0.7 of *F*_1_.

As reported in Table 6, OpenNRE-based models, trained jointly on a limited dataset of only 117 phenotype relations and 30 rare disease relations, show highly variable performance depending on architecture, input design, and pair selection strategy. CamemBERT generally outperforms ModernCamemBERT, and entity-based embeddings yield more balanced precision-recall than CLS-based representations. Passage-level inputs with LimitedPairs achieve the best *F*_1_ for rare disease relations (0.58), benefiting from local context and restricted candidate pairs, while FullText inputs improve recall for long-range links but reduce precision when allPairs are included. CNN-based encoders are inconsistent, often showing extreme precision-recall imbalances. Phenotype relations remain challenging, with the best neural configuration reaching an *F*_1_ of 0.42, underscoring the difficulty of capturing dispersed, long-distance temporal relations in clinical text, especially given the low amount of annotated training data.

These findings demonstrate the effectiveness of our LLM-based approach, which with few-shot examples substantially outperforms both neural and rule-based baselines for rare disease and phenotype relations with minimal annotation effort. While proximity-based rules offer interpretability, neural models typically require a substantial amount of annotated data, and both approaches struggle with long-distance, dispersed, and implicit relations. In contrast, our LLM-based approach robustly handles such complexities, including surface variations and temporal reasoning, providing clear advantages in low-resource clinical settings.

### Analysis of Relation Extraction on the i2b2 Clinical Concepts

As shown in Table 7, the evaluation of Qwen2.5 on the 2012 i2b2 clinical concepts demonstrates that our LLM-based approach generalizes effectively to a distinct clinical English dataset. While some studies [53] explored the fine-tuning and prompt-tuning of LLaMA and GatorTron variants on the original i2b2 task, reporting competitive performance, a direct comparison with our experiments is not straightforward since we adapt the corpus to our annotation scheme. These adaptations ensure consistency with our experimental framework but prevent a strict head-to-head comparison with prior LLM studies on the original corpus. Under this setting, Qwen2.5 achieves meaningful performance on 6008 annotated relations, with the best zero-shot configuration reaching an exact-match *F*_1_ of 0.4 and recall of 0.47, while few-shot prompting with only 10 examples improves recall to 0.53 while maintaining the same *F*_1_. These results are further validated by the LLM-as-a-judge evaluation. This performance is noteworthy, given the inherent difficulty of the i2b2 corpus, which is characterized by dense temporal annotations, implicit relations, and relatively low interannotator agreement [4]. Some errors of our LLM-based approach arise from the model’s tendency to infer temporal relations from contextual cues, occasionally producing plausible but incorrect links, while others may reflect omissions or inconsistencies in the gold annotations.

Overall, these results confirm that our LLM-based approach offers a robust and scalable solution for clinical TRE, even on challenging benchmarks, while remaining generalizable across datasets and languages in low-resource settings.

### Human Evaluation and Error Analysis of the Qwen2.5 Relation Extraction

Evaluating LLMs for information extraction remains challenging because traditional metrics are limited not only in their ability to capture semantic equivalence but also in their capacity to account for contextual and annotation-related variability. LLMs frequently generate synonyms, abbreviations, paraphrases, or normalized temporal expressions that are clinically correct yet do not exactly match reference annotations [70,71,74]. We therefore conducted a human evaluation of Qwen2.5, selected as the best-performing model, to further assess the quality of its predictions beyond exact-match and LLM-as-a-judge evaluations. This human evaluation confirms that a substantial proportion of extracted temporal relations, initially judged as incorrect, is in fact correct upon contextual review. Table 4 shows that Qwen2.5 achieves 0.71 of human-validated precision for few-shot phenotype relations and 0.85 for zero-shot rare disease relations, exceeding exact-match precision scores (Tables 2 and 3). Overall, human validation recovers up to 32 points with respect to exact-match precision (0.85 vs 0.53, rare disease diagnoses), with gains ranging from 6 to 32 points across configurations.

Our qualitative analysis highlights several sources of error that account for the gap between automatic metrics and human judgments. A recurring issue is annotation incompleteness, which underrepresents the temporal information in clinical narratives. For instance, in “crise d’epilepsie en 2013” (ie, epileptic seizure in 2013), the event and its temporal relation are annotated, but subsequent mentions like “8 à 9 crises par an en 2015 à 3 crises par an en 2018” (ie, 8 to 9 seizures per year in 2015 to 3 seizures per year in 2018) include coreferring event mentions (“crises,” ie, seizures) that are not annotated, leaving the corresponding temporal relations to “2015” and “2018” absent from the gold standard. Consequently, model predictions for these relations are penalized, despite being clinically valid. Similarly, for relations in “rechute de la maladie en 2012” (ie, relapse of the disease in 2012), the model is penalized because the referenced disease entity is not explicitly annotated as an event. These cases constitute annotation omissions rather than errors in the model’s temporal reasoning.

Other mismatches arise from annotation conventions that could, for instance, prioritize certain temporal cues over others or separately annotate individual mentions of entities. In our scheme, report dates are annotated only when they correspond to clinically salient events (eg, consultation, hospitalization, or procedure dates), while document metadata are excluded. However, the model sometimes extracts nearby temporal references such as header dates (“Paris, 18/02/2021”) or validation timestamps (“letter validated on 17/09/2019”) but fall outside the annotation guidelines. Additionally, variant disease mentions such as “oligo-arthrite juvénile idiopathique” (ie, Oligoarticular juvenile idiopathic arthritis) and “AJI oligoarticulaire” (ie, Oligoarticular JIA) denote the same underlying disease but are treated as distinct in the gold standard, penalizing correct temporal associations across synonymous nomenclature. When both “AJI” (ie, JIA) and “arthrite juvénile idiopathique” (ie, juvenile idiopathic arthritis) are annotated separately, the model often assigns the same date to both mentions, but only one relation is counted as correct under exact-match scoring.

Another source of error arises from the intrinsic complexity of clinical text, including nonstandard formatting and ambiguous temporal scope. For instance, in “Le 05/05/2003: Poids=42 kg ...” (May 5, 2003: Weight =42 kg ...), the date should be associated exclusively with the weight measurement and not with subsequent events mentioned in the text. However, the model frequently incorrectly propagates such narrowly scoped dates to later events, resulting in incorrect temporal relations. Similarly, constructions such as “depuis la dernière hospitalization de jour: ...” (since the last day hospitalization: ...) can lead to temporal references being extended beyond their intended scope. The model also struggles with relative temporal expressions such as “ce jour” (on this day) when contextual anchoring is ambiguous. In sections describing disease history, it sometimes assigns the report date to longitudinal events, even when explicitly instructed to return “None” if no temporal expression is present in such sections. The model also tends to favor selecting a concrete date over “None” when candidate lists include both options, resulting in false-positive temporal links.

Finally, some errors come from prompt specification gaps. While negated event mentions were excluded from the gold standard annotations, the model was not explicitly instructed to ignore them. Thus, it occasionally extracts temporal expressions linked to negated events, which are counted as errors under exact-match evaluation. Overall, our findings suggest that adapting annotation protocols and evaluation frameworks to better reflect the capabilities of LLMs, such as considering synonyms, coreferring events, and contextually valid temporal expressions, could provide more accurate assessments of model performance in clinical information extraction.

### Assessment of the End-to-End Prompt-Chaining Pipeline

The end-to-end prompt-chaining pipeline of Qwen2.5 integrates temporal expression extraction with subsequent normalization, illustrating the model’s ability to handle sequential clinical information tasks. In this setup, input configurations affect the extraction step, while normalization is consistently guided by a small set of 6 in-context examples applied to the previously extracted expressions. As shown in Table 8, rare disease events achieve higher combined *F*_1_-scores than phenotype events, suggesting that extraction is the primary driver of overall pipeline performance. The stability of results across input configurations indicates that prompt chaining effectively preserves dependencies between extraction and normalization, limiting error propagation. Even with a small number of in-context examples for normalization, the model can reliably standardize temporal expressions, highlighting that improvements in overall performance are mostly attributable to extraction quality. Most normalization errors involved partial dates such as “July” being normalized as “0001-07-01” instead of “2021-07-01,” due to missing guidance for such cases and the lack of access to the surrounding clinical context during normalization. Conversely, some cases showed strong normalization despite extraction mismatches, when the model directly produced a normalized date rather than its original textual form, which is counted as incorrect for extraction but correct for normalization.

Overall, the stability of results across configurations indicates that the prompt-chaining approach effectively maintains dependencies between extraction and normalization tasks.

### Carbon Footprint

The estimation of carbon emissions of our inference experiments highlights a substantial variability across models, corpora, and prompting strategies. As summarized in Table S7 in Multimedia Appendix 1, the TRE experiments on the i2b2 corpus using Qwen2.5 generated the highest carbon footprint. This is mainly due to the scale of the corpus, which contains 120 documents and 10,087 relations (Table 1), compared to our clinical corpus, which contains 42 documents with 356 phenotype relations and 70 rare disease relations. Qwen3 and DeepSeek-R1 produced lower CO_2_ equivalent (less than 1 kg), which could be due to the limitation of generated tokens we fixed to 2000 to prevent overly long reasoning of these models. As illustrated in Table S8 in Multimedia Appendix 1, few-shot prompting consistently increased carbon emissions compared with zero-shot prompting. The addition of rationale generation or given the list of possible dates seems to amplify costs due to longer input contexts and generated outputs.

### Limitations

The limited availability of annotated clinical data, which defines the low-resource settings targeted in our study, results in a relatively small evaluation dataset. Future work on larger annotated datasets would help further validate and generalize our approach.

Performance in temporal extraction is partly constrained by the dataset and task design. In our annotated corpus, nearly all temporal expressions associated with phenotypes or rare disease diagnoses are dates, with very few cases involving durations and almost none involving frequencies, limiting the assessment of model generalization to more diverse temporal phenomena. The fixed question formulation used for prompting further restricts coverage of complex or implicit temporal relations in clinical narratives, and annotation omissions or inconsistencies can penalize clinically valid predictions.

We evaluated multiple open-weight LLMs, but detailed qualitative error analysis and human validation were conducted only for the best-performing Qwen2.5. Additional experiments with smaller or more lightweight models may be needed, as they could offer lower computational cost for real-world deployment. We deliberately chose a zero- and few-shot prompting approach over fine-tuning to prioritize low-resource and easily deployable settings.

While the task of identifying and standardizing the date of an event may seem common, finding points of comparison with previous work remains difficult. We have tried to adapt the i2b2 corpus, which is widely used in the literature, to this task, but these adaptations do not allow for comparison with previous systems.

Carbon footprint measurements cover inference experiments only, excluding prompt refinement. These measurements were initially difficult to obtain reliably in the shared Ollama environment due to request queuing and dynamic model loading, though efficiency is a critical factor for clinical deployment.

### Conclusions

In this paper, we evaluated 4 open-weight, on-premises LLMs for TRE from French clinical narratives in a low-resource setting. We framed the task as question answering, combining zero- and few-shot prompting with prompt chaining for temporal normalization. Using a constructed and annotated real-world French clinical corpus, we compared model performance against rule-based and neural baselines through exact-match metrics, LLM-as-a-judge evaluation, and human validation. Results show that strong performance is achievable with few in-context examples for both phenotypes and rare disease diagnoses. Human validation of the best-performing Qwen2.5 predictions demonstrates that clinically correct temporal relations are often underestimated by traditional exact-match evaluations. We further demonstrate the generalizability of our approach on the English 2012 i2b2 corpus. Overall, carefully prompted generative LLMs can deliver high-quality TRE and normalization. Future work should explore annotation and evaluation frameworks better aligned with the strengths of LLMs to enable more accurate and clinically meaningful assessments.

## Supplementary material

10.2196/95198 Multimedia Appendix 1 Further details about data statistics and experimental results.

## Abbreviations

NLP: natural language processing
TRE: temporal relation extraction
LLM: large language model
CNN: convolutional neural network
PCNN: piecewise convolutional neural network
