# Supplementary material for: Leveraging Generative Large Language Models for Temporal Relation Extraction From French Clinical Narratives: Prompt-Chaining Approach
Source: J Med Internet Res. 2026 Sep 30;28:e95198. doi: 10.2196/95198 (PMC13626197; doi:10.2196/95198)
Supplement: Supplementary file 1 [file jmir-v28-e95198-s001.docx]

## Data statistics

Table S1. Character-based distance between considered events and associated temporal expressions in test sets.

|  | **#rel** | **mean** | **std** | **min** | **5%** | **10%** | **25%** | **50%** | **75%** | **90%** | **95%** | **max** |
| --- | --- | --- | --- | --- | --- | --- | --- | --- | --- | --- | --- | --- |
| **Phen**^a^ | 273 | 793 | 1103.17 | 13 | 25.6 | 41.2 | 97 | 383 | 1097 | 1999.6 | 3070.2 | 7291 |
| **Phen (w/o DocTime Relations)** | 110 | 146.46 | 165.11 | 13 | 18.45 | 23.8 | 41.25 | 87 | 177.5 | 428.9 | 553.55 | 654 |
| **RD**^b^ | 66 | 523.12 | 1102.85 | 14 | 32.5 | 41.5 | 57.5 | 159.5 | 315.5 | 1839 | 2363.75 | 6415 |
| **RD (w/o DocTime Relations)** | 26 | 62.15 | 30.99 | 14 | 30.5 | 33 | 43.75 | 51 | 81.75 | 98.5 | 111.75 | 155 |
| **Clinical Concepts^c^** | 6008 | 1526.19 | 1943.81 | 9 | 31 | 46.7 | 137 | 727.5 | 2225.25 | 4278.5 | 5656.55 | 13281 |
| **Clinical Concepts**  **(w/o DocTime Relations)** | 4148 | 1418.16 | 1988.57 | 9 | 27 | 37 | 78.75 | 390 | 2172.25 | 4350 | 5666.15 | 13281 |

^a^ Phen refers to phenotype events

^b^ RD refers to rare disease diagnoses

^c^ Clinical concepts refer to i2b2 clinical events used in our experiments

Table S2. Character-based distance between considered events and associated temporal expressions in train sets.

|  | **#rel** | **mean** | **std** | **min** | **5%** | **10%** | **25%** | **50%** | **75%** | **90%** | **95%** | **max** |
| --- | --- | --- | --- | --- | --- | --- | --- | --- | --- | --- | --- | --- |
| **Phen**^a^ | 117 | 703.61 | 1285 | 19 | 36.8 | 50.2 | 83 | 216 | 846 | 1340 | 2698 | 7550 |
| **Phen (w/o DocTime Relations)** | 69 | 195.38 | 218.99 | 19 | 30.6 | 38.6 | 62 | 104 | 196 | 556 | 650.8 | 928 |
| **RD**^b^ | 30 | 465.8 | 810.35 | 25 | 35.6 | 45.5 | 80 | 184.5 | 388.75 | 980.1 | 2353.75 | 3345 |
| **RD (w/o DocTime Relations)** | 11 | 151.36 | 261.9 | 25 | 28.5 | 32 | 44.5 | 57 | 120.5 | 164 | 546 | 928 |

^a^ Phen refers to phenotype events

^b^ RD refers to rare disease diagnoses

^c^ Clinical concepts refer to i2b2 clinical events used in our experiments

## Results

Table S3. LLM-as-a-judge evaluation of zero-shot & few-shot relation extraction performance (Rare disease) in terms of Precision (P), Recall (R) and F-measure (F).

|  | | **LLama3.3** | | | **Qwen2.5** | | | **Qwen3** | | | **DeepSeek-R1** | | |
| --- | --- | --- | --- | --- | --- | --- | --- | --- | --- | --- | --- | --- | --- |
| **Zero-shot extraction** | | P | R | F | P | R | F | P | R | F | P | R | F |
|  | Text | 0.33 | 0.67 | 0.44 | 0.71 | 0.76 | **0.74** | 0.25 | 0.82 | 0.38 | 0.25 | 0.71 | 0.37 |
|  | Text +rationale | 0.35 | 0.79 | 0.49 | 0.57 | 0.6 | 0.58 | 0.52 | 0.55 | 0.54 | 0.57 | 0.58 | 0.58 |
|  | Text +givenDates | 0.38 | 0.7 | 0.49 | 0.62 | 0.7 | 0.66 | 0.31 | 0.78 | 0.44 | 0.13 | 0.91 | 0.23 |
|  | Text +rationale +givenDates | 0.42 | 0.74 | 0.54 | 0.69 | 0.67 | 0.68 | 0.66 | 0.58 | 0.62 | 0.72 | 0.57 | 0.64 |
| **Few-shot extraction** | | P | R | F | P | R | F | P | R | F | P | R | F |
|  | Text | 0.41 | 0.85 | 0.55 | 0.58 | 0.82 | 0.68 | 0.48 | 0.7 | 0.57 | 0.46 | 0.7 | 0.56 |
|  | Text +rationale | 0.57 | 0.74 | 0.64 | 0.72 | 0.73 | 0.72 | 0.74 | 0.61 | 0.67 | 0.78 | 0.65 | **0.71** |
|  | Text +givenDates | 0.33 | 0.69 | 0.45 | 0.53 | 0.71 | 0.61 | 0.6 | 0.79 | **0.68** | 0.32 | 0.68 | 0.43 |
|  | Text +rationale +givenDates | 0.76 | 0.71 | **0.73** | 0.57 | 0.79 | 0.66 | 0.6 | 0.62 | 0.61 | 0.75 | 0.54 | 0.63 |

Table S4. LLM-as-a-judge evaluation of zero-shot & few-shot relation extraction performance (Phenotypes) in terms of Precision (P), Recall (R) and F-measure (F).

|  | | **LLama3.3** | | | **Qwen2.5** | | | **Qwen3** | | | **DeepSeek-R1** | | |
| --- | --- | --- | --- | --- | --- | --- | --- | --- | --- | --- | --- | --- | --- |
| **Zero-shot extraction** | | P | R | F | P | R | F | P | R | F | P | R | F |
|  | Text | 0.44 | 0.77 | 0.56 | 0.51 | 0.8 | **0.62** | 0.14 | 0.75 | 0.23 | 0.32 | 0.67 | 0.43 |
|  | Text +rationale | 0.44 | 0.76 | 0.56 | 0.53 | 0.69 | 0.6 | 0.5 | 0.55 | 0.52 | 0.52 | 0.6 | 0.56 |
|  | Text +givenDates | 0.47 | 0.76 | 0.58 | 0.5 | 0.76 | 0.61 | 0.48 | 0.87 | **0.62** | 0.1 | 0.85 | 0.18 |
|  | Text +rationale +givenDates | 0.47 | 0.77 | 0.58 | 0.53 | 0.73 | **0.62** | 0.54 | 0.67 | 0.6 | 0.55 | 0.63 | **0.59** |
| **Few-shot extraction** | | P | R | F | P | R | F | P | R | F | P | R | F |
|  | Text | 0.48 | 0.73 | 0.58 | 0.49 | 0.69 | 0.57 | 0.47 | 0.61 | 0.53 | 0.42 | 0.23 | 0.3 |
|  | Text +rationale | 0.59 | 0.61 | **0.6** | 0.55 | 0.67 | 0.6 | 0.6 | 0.56 | 0.58 | 0.59 | 0.43 | 0.5 |
|  | Text +givenDates | 0.44 | 0.8 | 0.57 | 0.42 | 0.64 | 0.51 | 0.44 | 0.72 | 0.55 | 0.24 | 0.59 | 0.34 |
|  | Text +rationale +givenDates | 0.61 | 0.54 | 0.57 | 0.53 | 0.7 | 0.6 | 0.6 | 0.58 | 0.59 | 0.65 | 0.43 | 0.52 |

Table S5. Exact‑match evaluation of the end‑to‑end pipeline for temporal relation extraction and normalization (Rare disease).

|  | | **LLama3.3** | | | **Qwen3** | | | **DeepSeek-R1** | | |
| --- | --- | --- | --- | --- | --- | --- | --- | --- | --- | --- |
| **Zero-shot extraction** | | P | R | F | P | R | F | P | R | F |
|  | Text | 0.33 | 0.66 | 0.44 | 0.12 | 0.82 | 0.22 | 0.42 | 0.68 | 0.52 |
|  | Text +rationale | 0.34 | 0.76 | 0.47 | 0.52 | 0.56 | 0.54 | 0.53 | 0.54 | 0.54 |
|  | Text +givenDates | 0.37 | 0.68 | 0.48 | 0.29 | 0.76 | 0.42 | 0.11 | 0.85 | 0.2 |
|  | Text +rationale +givenDates | 0.4 | 0.71 | 0.51 | 0.17 | 0.57 | 0.26 | 0.7 | 0.56 | 0.62 |
| **Few-shot extraction** | | P | R | F | P | R | F | P | R | F |
|  | Text | 0.4 | 0.81 | 0.53 | 0.46 | 0.7 | 0.56 | 0.44 | 0.66 | 0.53 |
|  | Text +rationale | 0.57 | 0.74 | 0.64 | 0.74 | 0.61 | **0.67** | 0.74 | 0.62 | **0.68** |
|  | Text +givenDates | 0.32 | 0.68 | 0.43 | 0.59 | 0.77 | **0.67** | 0.3 | 0.64 | 0.4 |
|  | Text +rationale +givenDates | 0.76 | 0.71 | **0.73** | 0.6 | 0.62 | 0.61 | 0.75 | 0.54 | 0.63 |

Table S6. Exact‑match evaluation of the end‑to‑end pipeline for temporal relation extraction and normalization (Phenotypes).

|  | | **LLama3.3** | | | **Qwen3** | | | **DeepSeek-R1** | | |
| --- | --- | --- | --- | --- | --- | --- | --- | --- | --- | --- |
| **Zero-shot extraction** | | P | R | F | P | R | F | P | R | F |
|  | Text | 0.43 | 0.75 | 0.55 | 0.06 | 0.76 | 0.12 | 0.31 | 0.67 | 0.42 |
|  | Text +rationale | 0.43 | 0.74 | 0.54 | 0.61 | 0.57 | **0.59** | 0.54 | 0.62 | **0.58** |
|  | Text +givenDates | 0.42 | 0.7 | 0.52 | 0.25 | 0.74 | 0.38 | 0.1 | 0.8 | 0.17 |
|  | Text +rationale +givenDates | 0.44 | 0.71 | 0.54 | 0.5 | 0.63 | 0.56 | 0.52 | 0.61 | 0.56 |
| **Few-shot extraction** | | P | R | F | P | R | F | P | R | F |
|  | Text | 0.48 | 0.72 | 0.57 | 0.24 | 0.61 | 0.35 | 0.45 | 0.25 | 0.32 |
|  | Text +rationale | 0.58 | 0.6 | **0.59** | 0.52 | 0.59 | 0.55 | 0.6 | 0.43 | 0.51 |
|  | Text +givenDates | 0.43 | 0.76 | 0.55 | 0.42 | 0.69 | 0.52 | 0.23 | 0.58 | 0.33 |
|  | Text +rationale +givenDates | 0.58 | 0.51 | 0.54 | 0.59 | 0.57 | 0.58 | 0.65 | 0.43 | 0.52 |

Table S7. Carbon emissions (in grams) generated by all models during inference for temporal extraction and normalization on our clinical corpus, and for temporal extraction on the i2b2 corpus.

|  | **CO2 equivalent (in grams)** | |
| --- | --- | --- |
|  | Our clinical corpus | i2b2 corpus |
| **Llama3.3** | 2321 | - |
| **Qwen2.5** | 1584 | 15 740 |
| **Qwen3** | 824 | - |
| **DeepSeek-R1** | 530 | - |
| **Total** | 5259 | 15 740 |

Table S8. Carbon emissions (in grams) generated for each model and configuration during inference for temporal extraction and normalization on our clinical corpus, and for temporal extraction on the i2b2 corpus.

|  | | **LLama3.3** | | **Qwen2.5** | | | **Qwen3** | | **DeepSeek-R1** | |
| --- | --- | --- | --- | --- | --- | --- | --- | --- | --- | --- |
| **Zero-shot extraction** | | CO2 eq (in g) | | CO2 eq (in g) | | | CO2 eq (in g) | | CO2 eq (in g) | |
|  | | RD | Phen | RD | Phen | i2b2 | RD | Phen | RD | Phen |
|  | Text | 17 | 84 | 14 | 76 | 1033 | 30 | 77 | 11 | 24 |
|  | Text +rationale | 25 | 122 | 24 | 126 | 2481 | 25 | 55 | 10 | 37 |
|  | Text +givenDates | 17 | 105 | 29 | 84 | 1084 | 16 | 30 | 17 | 46 |
|  | Text +rationale +givenDates | 28 | 131 | 35 | 40 | 2625 | 38 | 55 | 10 | 35 |
| **Few-shot extraction** | |  | |  | | |  | |  | |
|  | Text | 42 | 407 | 50 | 136 | 1349 | 28 | 87 | 20 | 53 |
|  | Text +rationale | 50 | 531 | 50 | 277 | 2858 | 37 | 100 | 21 | 63 |
|  | Text +givenDates | 45 | 185 | 52 | 207 | 1364 | 13 | 85 | 21 | 64 |
|  | Text +rationale +givenDates | 52 | 474 | 78 | 301 | 2942 | 37 | 107 | 21 | 69 |

Phen refers to phenotype events

RD refers to rare disease diagnoses

i2b2 refers to i2b2 clinical concepts
